# Supplementary material for: Are carbon emissions trading and green financial instruments synergistic? -Comprehensive quantitative research based on content analysis
Source: PLoS One. 2024 Mar 7;19(3):e0298601. doi: 10.1371/journal.pone.0298601 (PMC10919601; doi:10.1371/journal.pone.0298601)
Supplement: S1 Data — (ZIP) [file pone.0298601.s001.zip › Data/地级市Z_F工作报告.zip!/║┌┴·╜¡/┤≤╟∞╩╨/└┤╫╘╣1⁄2╓┌║┼í╛┬φ┐╦╩2╛▌═°í┐_2012.docx]

　各位代表：

　　现在，我代表市人民政府向大会作工作报告，请予审议，并请市政协委员和列席会议的同志提出意见。

　　一、过去五年工作回顾

　　市八届人大一次会议以来的五年，是大庆可持续发展进程中极不平凡的五年。我们在市委的坚强领导下，全面贯彻落实科学发展观，深入实施“一二三四五”发展战略，致力于创建百年油田、壮大接续产业、建设宜居城市、构建和谐大庆，积极克服国际金融危机带来的影响，全市经济社会发展取得重大成就。

　　五年来，扩总量调结构，经济发展整体跨越。着力构建以石油为基础，以石化为主导，以现代农业、装备制造、新材料和新能源、高端服务业为支柱的“1+5”产业格局，经济综合实力显著提升。全市地区生产总值先后跃上2000亿元、3000亿元台阶，由2006年的1620亿元增长到2011年的3737亿元。非油经济比重由36%提高到52.8%，经济转型进入新阶段。大庆在全国地级城市综合实力排名中跃升到第11位，可持续发展能力居全国资源型城市前列。农业突破发展。增加值由47.5亿元增长到133亿元。加大玉米膜下滴灌等农田基础设施建设和科技推广力度，粮食产量由52亿斤增长到111亿斤，作为龙江西部旱区用不足全省6%的耕地产出了10%的粮食；大牲畜和禽类饲养量分别是2006年的1.5倍和2倍，牛奶单产水平全省第一，畜牧业增加值由21.9亿元增长到59.4亿元；棚室由1.2万栋增加到14万栋，时令果蔬产量由5万吨增加到50万吨，加快向东北一流的果蔬生产基地迈进。农业综合生产能力的整体提升，为农民多元增收、农村经济繁荣奠定了坚实基础。工业扩张发展。增加值由1352亿元增长到2997亿元。油化根基更加稳固，原油4000万吨持续稳产，天然气产量突破30亿立方米，规上石化工业增加值由90亿元增长到393亿元。地方工业高速增长，年均增速达到40%，增加值由152亿元增长到750亿元，战略性新兴产业规模和增速全省第一，地方工业挑起接续产业大梁。统计基数调高后的规模以上工业企业达到450户，主营业务收入超亿元工业企业由58户增加到176户，超10亿元工业企业由8户增加到17户，省级以上著名商标和名牌产品由60个增加到154个，企业竞争力明显增强。服务业升级发展。增加值由182亿元增长到534亿元，社会消费品零售总额由252亿元增长到694亿元。首个城市综合体万达广场建成投用，湿地旅游文化节和雪地温泉节连续成功举办，文化创意产业园成为国家级试验园区，新肇内陆港填补功能空白，商贸、旅游、文化、物流、金融和服务外包等产业全面升级，市民生活消费和企业生产需求得到更好满足，有力地拉动了经济、繁荣了城市。

　　五年来，转方式提效益，内生动力显著增强。坚持“好”中求“快”，促进经济增长由外部政策刺激向自主增长有序转变，发展速度、质量和效益同步提升。累计上缴中央财政2184亿元、省级财政436亿元，地方财政收入由85亿元增长到244亿元。减排化学需氧量1.2万吨、二氧化硫9000吨，万元gdp综合能耗下降20.7%。多元投资拉动力更加强劲。固定资产投资由385亿元增加到1049亿元，其中石油石化大企业投资由249亿元增加到414亿元，地方投资由127亿元增加到564亿元，产业项目投资由287亿元增加到651亿元，非国有投资由66亿元增加到300亿元，全市投资拉动经济年均增长7个百分点。产业项目牵动力持续增强。累计建设单项投资千万元以上产业项目1056个，其中超亿元281个、超10亿元42个、超30亿元16个、超百亿元8个。30万吨聚丙烯、伊利脱脂奶粉、博润玉米等845个项目投产投用，全面提升了产业规模层次。120万吨乙烯改扩建、30万吨聚丙烯二期、千万吨炼油、550万吨重油催化热裂解等项目加快建设，带动石化产能扩张；沃尔沃乘用车生产基地项目加快建设，带动汽车产业高点起步；忠旺铝材项目加快建设，带动新材料产业成长升级；北国之春梦幻城项目加快建设，带动旅游目的地城市进程提速；新华08国际石油资讯中心、联想科技城等项目加快建设，带动战略性新兴产业集群发展，接续产业形成了龙头牵动、名企引领、新兴崛起的整体态势。园区综合承载力大幅提升。高新区国内综合排名由34位上升到29位，在东北率先获批建设国家创新型科技园区；经开区和林源工业区开发面积达到11.5平方公里，装备制造、精细化工等产业项目加快集聚；德力戈尔、杏山、光明等各县区特色园区竞相发展。全市园区工业增加值占地方工业比重由42%提高到69%，园区成为产业承载的大平台、企业孵化的主战场、经济发展的增长极。

　　科技创新驱动力加速释放。科研院所和分支机构达到170家，科技成果荣获省部级以上奖励232项，累计转化科研成果1480项，国家级高新技术企业发展到92户，高新技术产业产值由442亿元增长到1500亿元，科技进步贡献率达到50%。科技成为升级传统产业、培育新兴产业的动力引擎和撬动经济发展的有力杠杆。 五年来，强功能塑品质，城乡建设全面升级。遵循“生态、自然、现代、宜居”理念，坚持系统规划、整城建设、纵深管理、开放开发，实施市级重点工程188项、完成投资1406亿元，形成了集中快速建设期，基本实现了由局部建设向整体开发、基础功能补位向综合功能提升、矿区城市向现代都市的历史性转变。城市空间格局拓展优化。强化规划设计，聘请国际国内名家名院，高标准编制完成基础性规划和专项规划310项，形成了完整的城乡规划体系。启动建设规划总面积366平方公里的庆南、庆北、庆东和庆西“四个新城”，建成区由159平方公里扩大到225平方公里，拉开了城市战略框架，留出了长远建设空间。现代交通体系整体构建。全市人民多年期待的机场建成通航，国家高速路网布局中的大广高速大庆段贯通市域南北，肇源新港实现江海联运，铁路客运东西两站和城际客运专线加快建设，萨大路、西一路、龙凤湿地大桥、公路客运枢纽等39项重点项目竣工投用，为大庆融入更大区域经济循环搭建了水陆空立体交通大通道。市政基础设施更加完备。建设东城水库水厂、东城热源等重点市政基础设施项目34项，新建改建城区供热供水供气管网2150公里，集中供热普及率全省第一；建设垃圾和污水处理厂15座，城市生活垃圾无害化处理率和生活污水集中处理率分别由80%、70%提高到87%、92%，在全省率先实现县县有生活垃圾处理场和污水处理厂。公共服务功能显著提升。城市规划展示馆、市博物馆、西城体育馆、市中医院等一大批项目建成投用，奥林匹克公园、世博会石油馆迁建、黑鱼湖国际艺术村等重点项目加快推进，中央党校、国家行政学院、国防大学在大庆设立教学基地。绿色生态持续优化。深入推进“植树、复草、治水、净气、降噪”工程，有林面积由277万亩增加到350万亩，城区绿化面积由4751公顷增加到8942公顷，全市森林覆被率由8.7%提高到11%，建成区绿化覆盖率由36%提高到45%；综合治理三永湖、兰德湖等湖泊19个，黎明河由原来的排污干渠成为靓丽滨水景观和市民休闲佳所。以绿为底色，以水为特色，以净为本色，大庆的生态环境更富魅力。城市管理水平明显提高。城乡环境“十项”综合整治深入推进，3000个网格覆盖社区村屯，2.1万个视频探头遍布厂矿小区、公共场所、重要部位，数字化智能监控系统整体升级，实现了管理区域由城区向农村延伸，管理方式由传统向现代提升，管理机制由突击治理向常态长效转变。大庆荣获全国文明城、国家卫生城、国家环保模范城，成为同时拥有“三城”品牌的全国七个地级城市之一。

五年来，惠民生办实事，人民生活大幅改善。加快构建“五和”社会、创造“五新”生活，满足基本民生、建设小康民生、发展现代民生。城镇居民人均可支配收入由14791元增长到22500元，农村居民人均纯收入由3610元增长到9300元，城乡居民收入比由4.1：1缩小到2.4：1，提前达到全面小康社会标准。买车、换房、度假等趋优消费持续旺盛，占居民消费支出比重由16.7%提高到31.6%，大庆在全国地级城市居民富裕指数排名中列第41位。教育更加均衡。公办幼儿园由139所增加到232所，全市学前三年入园率达到80%，高于全省平均水平33.6个百分点；义务教育标准化学校发展到212所，“双高普九”实现全覆盖；高考成绩连续10年保持全省领先，市区学生享受优质高中教育比例由74%提高到85%，位居全国前列；中等职业学校达到15所，累计培养各类技能型人才7万人；7所高校在校生达到6.7万人，高校数量和在校大学生人数居全国地级城市前列。医疗更加优质。5所三甲医院领衔、8所市级医院辐射、24所县区医院带动、681所基层卫生机构承载的医疗卫生服务体系初步形成，三级医院领办社区卫生服务模式全国推广，心脑血管病、眼病、心理精神卫生等8大医疗专科整体水平处于全省前列，与卡尔加里医学院、北京协和、解放军301等国内外53所名院626位名医开展合作共建，市民足不出市就有机会有条件享受到全国一流的诊疗服务。文化更加惠民。文化基础设施全面完善，482个标准农家书屋、58个乡镇综合文化站、339个社区文化活动场所、550个文化信息资源服务点覆盖城乡；文体事业成果丰硕，《铁人轶事》、《鹤鸣湖》等文艺精品不断涌现，荣获国家级以上文化奖项190项，获得省级以上体育赛事金牌135枚，文化影响力和竞技体育水平显著提升；“书香大庆”、“十百千万”全民文化和健身等活动广泛开展，群众文体生活丰富多彩。保障更加有力。持续扩大就业，在全省率先实现临近低保人群和残疾人就业援助，城乡新就业21万人次，城镇登记失业率控制在4.5%以内；持续推进社保提标，在全省率先实现全民医保，新农合参合率达到99%，“新农保”、“城居保”启动实施，“五七工”、“家属工”纳入养老保险，“五险”扩面111万人次，归集住房公积金300亿元，在全国率先建立城镇“十位一体”和农村“六位一体”低保综合救助体系，市区最低工资和城乡低保救助标准全省最高；持续强化为老服务，新建市第二福利院和29所县乡敬老院，扶持发展民办养老机构42家，新建社区老年人日间照料室201个，初步形成公办引领、民办补充、居家为主的社会化养老服务体系；持续优化公共交通，新增公交线路里程136公里、公交车320台，新投放出租车3500台，市民乘车难问题得到有效缓解；持续改善住房条件，242万平方米主城区棚户区改造基本完成，144个城市老居住区旧貌换新颜，14.2万户农村泥草房改造“八年任务四年完”，地方和大企业共同建设回迁安置房、经适房等保障性住房929万平方米，城镇人均住房建筑面积由35.5平方米提高到40.1平方米，农村住房砖瓦化率由46.2%提高到82.5%，城乡居住环境明显改善。 五年来，增活力促合作，改革开放纵深拓展。坚持用改革的办法解决深层次矛盾，通过开放的路径拓展更大发展空间，最大限度地释放发展活力。重点改革扎实推进。市、县区两级政府机构改革顺利完成，驻外办事机构改革基本完成，审批制度改革成效明显，行政管理体制更加顺畅；医药卫生体制改革取得阶段性成果，城乡医疗服务水平切实提高；扁平管理、警力下沉、综合执法，公安警务体制改革走在全国前列；组建新闻传媒集团和文化集团，文化体制改革取得重要突破；创新政府投资项目招投标机制，资金使用效率明显提升；成立市属公交公司，公交运营体制向地企共办转变。经济合作全面加强。中俄原油管道漠大线建成输油并在庆报关结算做销，大庆成为集原油生产、加工、贸易于一体的国家战略能源基地。全市有进出口业绩企业突破100户，进出口贸易总值由4.4亿美元增长到21.6亿美元。新增同禹傲思等上市企业12户、达到19户，累计融资100亿元，境外上市企业户数居全省首位。联想等在庆投资的国内外500强企业达到38户，五年累计引进到位资金786亿元。对外交流日益活跃。与俄罗斯秋明等104个境外城市和地区建立稳定的经贸合作关系，与广西南宁、韩国忠州等12个国内外城市结为友好城市，先后有75位外国政要和驻外使节来庆访问考察，《欧洲时报》等36家海外华文媒体多次宣传推介大庆。在16个国家设立驻外经贸联络处，发起建立“连理同庆”城市旅游联盟，成功举办中国经济50人论坛、大庆文博会等全国性区域性论坛会展。大庆入选中国最具投资潜力城市50强，成为世界特色魅力城市200强之一。

　　五年来，夯基础保平安，社会管理不断创新。完善“党委领导、政府负责、社会协同、公众参与”的社会管理格局，建立“两保四大”工作机制。基层基础更加扎实。推行“居站分设、选聘分开”，社区全部建立社会工作站，承接劳动、就业、社保等67个服务事项，群众办事更加便利；全面完善社区和行政村的办公、活动场所，建设标准全省领先；采取奖励、补贴或委托管理等方式，扶持社会组织发展养老、文化、医疗等服务，社会公共服务供给能力不断增强；开通社区服务呼叫中心，加盟商家1.5万个，居民足不出户就可享受到家政、配送、保健等生活服务，大庆成为全省和谐社区建设示范市。在全省率先推行“居住证”管理制度，流动人口更好地享受到市民待遇。深入开展拥军优属、拥政爱民工作，大庆连续五届荣获“全国双拥模范城”。矛盾防范调处及时有效。完善“六调联动”工作体系，成立市、县区两级群众来访调处中心，创建“四级”社会和群众工作网络，全面推广“五进七讲”工作法，实行领导干部接访包案、信访代理和重大事项社会稳定风险评估等制度，各级人民调解组织调处各类矛盾纠纷13万件次，源头防范能力切实增强。社会始终保持平安稳定。扎实推进“平安大庆”建设，八类主要刑事案件下降66.6%；保持“八大领域”安全监管强势，整体提高人防、物防、技防和联防水平，安全生产事故下降25.1%；深入开展“打四黑除四害”专项行动，市场秩序更加规范；组建城市应急指挥中心和12个专业应急救援中心，累计帮助群众解决“急难险特”事项2.6万个，快速反应和应急救援水平明显提高。大庆入选中国幸福城市社会管理创新最佳实践案例。

事实证明，过去五年是大庆建设史上又一个经济社会发展最快的五年、城乡面貌变化最大的五年、人民生活受益最多的五年、创业创新成效最好的五年。五年取得的发展成就，得益于党中央、国务院特殊关怀、深切厚爱，得益于省委、省政府和中油集团高度重视、全力支持，得益于市委统揽全局、坚强领导，得益于人大政协有力监督、积极帮助，各民主党派、工商联、无党派人士和群团组织建言献策、广泛参与，得益于中直石油石化大企业鼎力相助、携手共建，得益于广大干部群众求真务实、团结奋斗。在此，我代表市人民政府，向全市人民，向中省直单位、高校、驻庆解放军和武警官兵，以及所有关心支持大庆发展建设的同志们、朋友们，表示崇高的敬意和衷心的感谢！

　 在总结五年发展成绩的同时，我们也清醒地认识到存在的问题和差距。主要是，接续产业层次和科技创新水平相对不高，经济发展方式需要进一步转变；城乡公共设施建设不够均衡，区域中心城市承载力和辐射力相对不强，现代城市功能需要进一步提升；城市开放度和经济外向度相对较弱，参与更大区域更广领域的国内国际交流合作需要进一步突破；部分城乡居民生活质量相对较低，住房、就业、看病、上学等方面存在的困难需要进一步解决；个别部门和公务人员的服务意识不强、办事效率不高、工作作风不实，政府效能建设需要进一步加强。 二、今后五年目标任务和2012年主要工作

　　刚刚闭幕的市八次党代会，作出了建设现代化国际化城市的重大战略决策，标志着大庆承前启后进入新的发展时期，继往开来踏上新的奋斗征程。

　　蓝图绘就，宏伟目标激励我们奔向未来。市委确定了建设现代化国际化城市“三步走”战略，到2015年实现全面小康，现代化取得实质进展，地区生产总值突破6000亿元，产业、市政、人文、体制基础更加坚实；到2020年基本实现现代化，打牢国际化基础，常住人口达到350万，建成区达到400平方公里，地区生产总值突破万亿；到2050年现代化达到更高水平，初步实现国际化，常住人口达到550万，建成区达到500平方公里，各领域发展与国际交融。在这一宏伟目标引领下，大庆发展将加速向现代转变、向高级阶段跃升，经济会越来越繁荣发达，社会越来越文明和谐，城市越来越美丽宜居，生活越来越幸福安康，必将极大地鼓舞当代大庆人再创新业的士气，广泛凝聚全市各方再续辉煌的力量。

踏上征程，综合优势助推我们阔步前行。经济全球化、区域一体化愈发明显，国际间、区域间、城际间交流合作日趋活跃，我们有参与全球经济循环的更多机会；国家大力支持东北老工业基地振兴，省里强力推进“八大经济区”和“十大工程”，希望大庆在全省发挥领跑作用，我们有转型发展的有利环境；大庆资源禀赋丰厚、油化根基稳固、接续产业多元、市政设施完备、人力资源充足、服务环境优良，我们有集聚要素的良好经济生态；大庆精神、铁人精神传承弘扬，开放、包容、时尚、文明的城市文化彰显魅力，我们有创新业创大业的持续动力。只要抢抓机遇，乘势借力，就会构筑起可持续发展的新优势。

　　理性思考，困难矛盾需要我们全力攻坚。对照建设现代化国际化城市指标体系，服务业比重偏低、建成区规模偏小、跨国公司分支机构偏少、经济外向度偏弱，缩小差距、实现目标的压力加大；伴随城市规模的持续扩张、经济活动的日益活跃、人口结构的加快变化、民生需求的更趋多元，创新城市管理、加强社会建设的压力加大；各地竞相赶超，要素、人才和市场的争夺愈加激烈，大庆实现更高层次发展的压力加大。我们一定要清醒认识、直面挑战，勇于善于破解矛盾问题，推动发展不断迈上新台阶。

　　使命在肩，创新创业要求我们奋发有为。建设现代化国际化城市，是市委作出的重大决策和历史抉择，是全市人民的殷切期盼和共同福祉。完成这一光荣而艰巨的任务，要求我们必须解放思想，敢于超越权威、超越前人、超越自我，不回避差距、不惧怕竞争，以更大的决心和勇气奋力前行；必须“跳出大庆看大庆、站在高处看大庆、分析对比看大庆”，不求一时之功、不限一域之争，用战略思维谋划发展；必须“负责任地干事、干负责任的事”，励精图治，脚踏实地，一步一个脚印地去实践、去创造、去奋斗，每项工作都有新进展，每年都有新建树，努力把宏伟蓝图变成美好现实。

各位代表，今后五年我们要全面落实市八次党代会精神，紧紧围绕建设现代化国际化城市大方向，深入实施“一二三四五”发展战略，致力于建设富裕大庆、宜居大庆、和谐大庆、文明大庆、智慧大庆、活力大庆，以科学发展为主题，以转变经济发展方式为主线，加快项目建设力促增长、完善城乡功能力促提升、扩大内外合作力促开放、统筹改善民生力促均衡、加强社会管理力促和谐，建成全面小康社会，开创现代化国际化城市建设的新局面。今后五年，地区生产总值年均增长12%以上；城镇居民人均可支配收入和农村居民人均纯收入分别超过40000元和19000元，综合实力位居全国前列。按照市委总体部署，着力推动经济社会发展实现“六个重大突破”：　——围绕建设富裕大庆，推动产业发展实现重大突破。服务创建百年油田，保持油田稳产形势、加强持续稳产基础、提升开发整体水平，筑牢大庆经济发展根基。提升“四大基地”，加快发展现代农业，标准化推动种植业增效，集约化带动畜牧业提质，产业化牵动棚室经济扩张，农业增加值实现倍增，建成龙江西部现代农业示范区。挖掘优势、抢占高端、集群发展现代工业，延伸增值做深石油化工，集聚配套做大装备制造，龙头带动做强汽车产业，高点切入做优新材料和新能源，瞄准前沿做精生物和生命科学等产业，构筑起传统工业升级转型、新兴工业加速崛起的新优势。创意引领、关联融合、跨越发展现代服务业，形成区域金融、商贸、会展、资讯中心，建设旅游目的地城市，现代服务业增加值年均增长20%以上。全面完成“5·10·15”计划，打造形成“四个产业聚集区”，初步建立起高科技、低碳化、开放型的现代产业体系。

——围绕建设宜居大庆，推动城乡建设实现重大突破。坚持“生态、自然、现代、宜居”理念，强化顶层规划设计，以世纪大道为中轴打造城市带状中心区，以“四个新城”为依托打造分工合理的特色功能区，以小城镇和示范村为牵动打造梯次衔接的外围综合区，加快构建城区中心辐射、四县卫星组团、新型城镇环拥的空间格局。升级立体交通，谋划建设至绥化、黑河的北向铁路和高速公路，形成区域交通“十字”枢纽，开辟国际航线、建设干线机场，扩建肇源新港，构建起集国际航空、城际快轨、江海联运及市内快速通道于一体的现代交通网络。提升市政设施，重点谋划建设大型水源、热源、电力等重要基础设施，整体建设城市地下综合管廊系统，打牢长远发展的基础保障。塑造特色风格，开辟城市运河，打造平原森林，建设生态景观，融入文化内涵，提升城市现代化管理水平，基本建成国家园林城市和国际人居优善城市。

　　——围绕建设和谐大庆，推动民生改善实现重大突破。健全“六大”体系，实现学有优教、劳有所得、病有良医、老有颐养、住有宜居。提升居民收入水平，鼓励创业，扩大就业，城镇居民人均可支配收入年均增长12%以上，农村居民人均纯收入年均增长15%以上；提升公共服务水平，教育、卫生、科技等社会事业在全省率先实现现代化，均衡优质便民的普惠型公共服务覆盖城乡；提升社会保障水平，低保综合救助能力持续提高，城乡养老保险全覆盖，住房保障和社会化养老服务进入全国先进行列；提升社会管理水平，建立科学的社会管理模式、健全的公共安全体系、规范的市场经济秩序，社会更加公正公平，生活更加平安和谐，群众幸福感进一步增强。

——围绕建设文明大庆，推动文化繁荣实现重大突破。塑造核心价值观，大庆精神、铁人精神成为城市的人文根脉，转化为引导全市人民投身建设现代化国际化城市的文化力量。提高全民文化素养，倡导爱国守法、明礼诚信、团结友善、勤俭自强、敬业奉献的道德和行为规范，培育阳光平和、开放包容的公众心态，形成现代文明的社会生活方式，促进人的全面现代化。提供优质文化服务，文化设施健全完善开放共享，文化活动活跃城乡深入人心，文化精品不断涌现彰显特色，文化服务更好满足多元需求，全市公益性文体设施达标率超过90%。引领扩大文化消费，创意催生特色文化产品，专业运作文化市场，培育打造文化产业集群，文化产业增加值年均增长30%以上，成为接续产业的重要支柱。

　　——围绕建设智慧大庆，推动创新引领实现重大突破。加快产业智能化，大力促进科技创新和科技成果转化，加速信息技术与农业、工业、服务业深度融合，启动建设云计算中心、移动互联中心和物联网中心，基本建成国家新一代信息技术产业示范基地。加快管理智能化，推动新兴技术与政务管理、市政管理、社会管理紧密结合，全面构建政务运行、市政设施运营、公共安全监管、应急指挥等重点领域的智能化管理系统，提高管理效率效能。加快便民服务智能化，建设就医就学就业和社保低保医保等便民服务智能化平台，提高电子商务、社区服务、家居生活等信息化水平，市民能够更好地享受到“一卡通”、“一站式”、互动性、高效率的智能服务。 ——围绕建设活力大庆，推动改革开放实现重大突破。着力解决影响和制约经济社会发展的深层次矛盾，推动政务运行更加高效、要素流动更加通畅、非公经济更加活跃、发展环境更加优良，构建有利于现代化国际化城市建设的体制机制；着眼全球紧盯东北亚，扩大石油石化、机械装备、资讯外包等优势领域经贸合作，加速壮大外向型生产、服务和贸易企业群体，建设国际航空港等功能平台，更广泛地参与区域性国际性经济合作，在庆投资、设立区域总部或分支机构的国内外500强企业超过100家，进出口贸易总值翻两番，经济外向度提高5个百分点。全方位深化对外文化、教育、医疗、体育、科技等领域交流合作，提升全国性和国际性重要会展赛事的举办承办能力，城市开放度和影响力显著增强。

　　各位代表，2012年是实施“十二五”规划的承上启下之年，是建设现代化国际化城市的开局起步之年。经济社会发展的主要预期目标是：全市地区生产总值增长12%，地方财政收入增长22%，固定资产投资增长35%，城镇居民人均可支配收入超过25000元，农村居民人均纯收入突破10000元，万元GDP综合能耗下降3.5%。

　　（一）抓好油化服务。坚持“四共”方针，全力以赴搞好油化企业生产生活服务。突出保障4000万吨稳产。重点维护油田产能秩序，严厉打击偷盗油气和电力设施等违法犯罪行为；整治产能区生产环境，加大违建和占压油气管线建筑拆除力度，消除外在安全隐患；保障油田勘探开采用地，协助做好占地、补偿等相关工作，为产能建设提供强力支持。服务石化项目顺利推进。大力支持120万吨乙烯改扩建、30万吨聚丙烯二期、千万吨炼油等项目按计划推进，确保年内建成投产。积极协助企业谋划争取“十二五”重点项目，力争在扩大炼油规模、推动石化延伸增值等方面有新进展。统筹和谐矿区与现代城市同步建设。支持创业大道升级、龙南医院扩建、商厦地区综合改造，促进矿区基础设施和服务功能更加完善；支持矿区开展综合治理，绿化生产区，美化居住区；支持创业城、石化会战园等居住新区建设和老居住区改造，进一步改善职工生产生活环境。

　（二）抓好产业项目。深入实施“三百”行动计划，服务企业更好发展做优存量，突出产业项目建设做大增量。新建续建千万元以上产业项目367个，其中超亿元186个、超10亿元49个，力争开复工率和投资强度再创新高。建好“四大基地”和棚室经济为重点的现代农业项目。推进旱田节灌和水田灌区项目，全力建好30个现代农业示范区，新增膜下滴灌和喷灌110万亩，力争粮食增产10%。着力打造四季青温泉果蔬、大同青提、红岗花卉等10个特色基地，全市新建棚室6万栋、总量达到20万栋。加快建设6000吨提子醋等加工项目，大力推进冷链仓储、物流中心、农超对接等服务配套项目，扩大棚室经济综合效益。深入推进养殖方式“三进三退”，新建改建牧场和小区140个，奶牛肉牛集中饲养率提高到70%。全市农业增加值增长15%。建好立市龙头和战略新兴为重点的地方工业项目。加速推进550万吨重油催化热裂解、50万吨C综合利用等在建石化项目，谋划建设聚烯烃、合成橡胶、液态化工等延伸增值项目，拉长石化产业链条；全力推动沃尔沃乘用车生产基地项目尽快形成整车生产能力，跟进引建汽车零部件配套项目；启动建设低碳光伏产业园，同步开工电池切片、光伏组件、光伏发电等全产业链项目，大力推进忠旺铝材、福瑞邦生物制药、三峡风电等项目，带动战略新兴产业更快发展。全市地方工业增加值增长30%以上。建好名企引领和高端业态为重点的服务业项目。坚持“随着城市进程走、迎着生活需求上、围着生产服务抓、跟着深度创意谋”，生产服务业与生活服务业并重，现代服务业与传统服务业并举，新建续建服务业项目100个以上。加快建设唐人中心等城市综合体和红星美凯龙、奥特莱斯等高端名店，带动形成城市商业商务和生活服务中心区，在满足市域需求的同时提升域外辐射带动能力，社会消费品零售总额增长20%；加快建设北国之春梦幻城、龙凤湿地公园、阿木塔风情园等旅游项目，引进北京秦龙等旅游企业，高水平办好第五届湿地旅游文化节，旅游总收入增长30%，打造全季、全域、全业和全民旅游的新格局；加快建设文化创意产业园、新华08国际石油资讯中心、黑鱼湖国际艺术村等文体项目，搞好大型场馆运营，推动发展创意设计、演出赛事、文博会展，文化产业增加值增长30%；加快建设太阳升综合物流园、龙凤昌升物流园等项目，完善提升新肇内陆港，规划建设高新区物流园，启动建设林甸、大同农产品果蔬物流中心，物流业增加值增长20%；加快建设金融产业园区等金融项目，引进中信、浦发等金融机构和外资银行，运作发行企业债券，金融业增加值增长25%；加快建设服务外包产业园，扩大石油工程技术服务、动漫设计和软件开发等高附加值业务比重，新增服务外包企业30户以上，营业收入突破80亿元。全市服务业增加值增长20%。建好基础承载和产业关联为重点的园区项目。以省市共建园区为牵动，加快推进全市16个园区水电路气讯、铁路专用线等基础设施建设，支持县区特色园区晋升省级园区。高新区和经开区突出建好联想科技园、生命科学产业园、大学科技创新园、高端装备园、绿色汽车产业园，林源工业区围绕建设550万吨重油催化热裂解，加快项目集群落地。全市园区工业增加值占地方工业比重达到70%。建好成果转化和企业孵化为重点的科技项目。推动政研校企协同创新，努力增强科技创新对产业发展的驱动力，促进“原字号”向“产业链”、“粗加工”向“高精尖”升级，建设国家杂粮工程技术研究中心、国家级技术转移示范机构，抓好高新区国家创新型产业集群试点，提升科技园、孵化器、中试平台等功能，推动科技成果产业化。全市转化省级以上科技成果超过100项，新增国家级高新技术企业10户，高新技术产业实现增加值550亿元。 （三）抓好城乡建设。坚持建管并重，进一步夯实市政基础。推进新城开发老区改造。庆东新城高起点开发湿地周边区域，完成青龙山地区基础设施配套；庆南新城重点推进三个组团基础设施、城区功能、生态环境提档升级；庆西新城加快启动让通线以西区域规划设计，整体建设富强地区；庆北新城重点规划启动北国之春梦幻城二期，加快兰德湖周边区域规模开发。强化城市重要节点、主要干线两侧破旧房屋整理和沿街立面整修，继续推进老居住区整体改造，实现公用设施配套、功能完善和环境优化。推进重点工程建设。实施好规委会确定的94项城建重点项目。加快建设铁路东西两站、龙永路改线等交通道路项目，实施北城热源、东城区给排水管网改造等“三供两治”项目，建好国际会议中心、西城区青少年科技活动中心、国家矿山公园等功能性项目，开发建设月亮湾、阿斯兰小镇等高品质居住新区。推进新型村镇建设。高起点建好11个新型小城镇和16个示范新村，推动近郊镇撤村变居、撤镇变街，整合国营牧场建设现代居住新区，加快推进连环湖、四季青、新站、朝阳沟、高台子等重点镇建设，同步跟进基础设施、公共服务、环境治理，培育壮大立镇兴村产业，确保一年见成效、两年大变化、三年成规模。推进生态环境建设。建好城市绿洲广场、未来广场、青龙山广场，提升休闲功能；搞好湖泊治理，建设万宝湖、三永湖、黎明湖“三湖景观栈道”，打造亲水滨水景观；新建颐翠园等片状森林3000公顷，绿化中八路等沿线193公里，城区植树1000万株，扩大绿量覆盖；加快百万亩沙地人工林项目建设，完成人工造林和封沙育林8万亩，改善沙区生态环境。加强现代城市管理。持续开展城乡环境“十项”综合整治，提高“门前五包”履约率，推行“六进网格”，新增城区视频监控探头1000个，城区保洁、清雪等机械化作业面积达到70%以上。全力做好迎接国家卫生城市复检工作，巩固提升“三城”联创成果。

（四）抓好民生实事。群众在我们心中有多重，我们在群众心中就有多重。保障和改善民生是政府工作的头等大事，要继续压缩一般性财政支出，增加民生方面的专项支出，进一步提升公共服务水平，解决好百姓关注的大事、要事、急事、难事，让群众得到更多实惠。促进教育均衡。顺应群众期盼，努力办人民满意教育。高标准改造新建中小学校120所、农村寄宿制学校11所，整体迁建四中和二十三中，启动建设实验中学二部，建成特殊教育发展中心；深入实施校园安全工程，强化校车秩序管理，对农村寄宿制学校食堂和农村中小学供暖给予补贴，加强校内食堂和校外“小饭桌”卫生防疫监管，保证孩子上学平安、住得暖和、吃得健康；加快建设职教园区和大型公共实习实训基地，培养多层次专业技术人才，促进职业教育与产业嫁接融合发展；支持高校提升教学科研水平，推动高等教育现代化建设。促进文化惠民。实施“铁人故事和油田创业历史进校园工程”，以图书画册、动漫影视、网络展馆等形式深化青少年人文素质教育；建成奥林匹克公园、市图书馆、世博会石油馆等项目，改建新建标准社区和行政村综合文化活动场所100个，公共文化活动场馆和乡镇文化站全部免费向群众开放；办好“辉煌大庆”北京文化艺术周，开展“百湖之春”等文化活动，丰富群众文化生活。促进医疗优质。加快推进市妇女儿童医院、市三医院扩建等重点项目建设，进一步改善乡镇和村医疗卫生条件，继续完善社区卫生服务机构功能，全面加强“六个对接”，深入推进与国内外名院名医合作，重点建好远程会诊系统，为群众提供更加便捷、质优、价廉的医疗服务。促进保障提标。城镇职工和城镇居民基本医疗保险政策性支付比例达到90%和70%以上，新农合人均筹资水平达到290元。进一步提高城乡低保标准和财政补助水平，农村五保对象集中供养、分散供养标准每人每年分别提高860元和570元。将长期义务献工无报酬的社区党支部书记、社区居委会居民小组长误餐费纳入财政预算，每人每月补助200元。

倾力办好十件民生具体实事。一是幼儿入园。结合新区开发、老区改造和小城镇建设，新建改建城乡公办园90所，在园幼儿占全市入园幼儿总数的比例达到65%。二是市民出行。优先保障未通公交的偏远区块、运力不足的重点区域，新增公交线路6条，调整增投公交车246台，新投放出租车1000台。三是小区环境。对1985年以前建成的团结路、中林街、景园等55个老居住区进行全面改造，完善功能美化环境。四是农民看病。新改扩建乡镇卫生院12所、村卫生室157个，所有村卫生室全部纳入国家基本药物制度管理，乡镇卫生院医务人员工资全部纳入财政预算管理，积极化解乡镇卫生院债务负担。五是大学生和退转军人就业。通过辟建毕业生就业见习基地、落实“三支一扶”项目等多种渠道，支持退转军人自主创业、依据政策安置等有效举措，做好大学生和退转军人就业工作。六是低收入群体住房。新建经济适用住房6.9万平方米，续建公共租赁房8.5万平方米，有效解决棚户区改造回迁居民、城市低收入家庭和新就业大学毕业生住房问题。七是中小企业融资。组建肇源农村商业银行，新增小额贷款机构10家、上市企业2户，进一步解决好中小企业融资难问题。八是社会化养老。基本实现“新农保”、“城居保”全覆盖，新增民办养老机构7家、城乡老年人日间照料室40个，加快构建社区15分钟为老服务圈。九是物业供热管理。规范物业企业服务项目、收费标准，推行供热企业数字化在线监控，提高服务质量。十是群众文化休闲。打造6个文化广场、5条文化街区、10个滨水休闲区，拓展市民休闲空间。改建新建四县和大同有线电视发射站，在全省地级市率先开通高清数字电视频道，提高城乡居民收视质量。（五）抓好社会管理。突出重点人群服务管理。坚持以人为本、服务为先，围绕“十类特殊人群”，全面推行“居住证”管理制度，实现流动人口市民化待遇；建立社区矫正对象和刑释解教人员过渡性安置中心，减少重新犯罪；完善流浪乞讨人员救助站功能，做好救助、管理和解救工作；开展精神残疾群体防治与康复社会化服务，健全监测救治、服务管理等机制；救助智残人员家庭，保障基本生活；推广“96760”老年应急呼叫系统，提供应急、保健、家政等服务。突出重点矛盾防范化解。围绕新形势下容易引发信访和影响社会安宁的征地拆迁、劳资矛盾、土地纠纷、文化冲突、违法犯罪、流动人口管理、突发公共安全事件、社会心态失衡、复杂场所整治、刑释解教人员继续教育等十个方面问题，深入开展普法宣传教育，落实重大事项社会稳定风险评估机制，健全“四级”社会和群众工作网络，发挥“六调联动”综合作用，引导社会组织参与社会管理。推进“百项服务”进社区，推动社会工作站向乡镇和村延伸，全面加强社区社会组织规范管理，力争做到需求满足在基层、诉求回应在基层、矛盾化解在基层。强化互联网安全监管，健全完善重大事件网络舆情快速反应和应急处置机制，依法规范网络信息传播。突出重点领域安全监管。围绕食品、药品、交通、建筑市场、消防、生产、校园、矿区等“八大领域”安全，着力增强安全防范和应急处置能力，集中开展饮水安全、食品安全、药品安全等专项治理，确保安全形势持续稳定；深化“打四黑除四害”行动，严厉打击侵财涉众及暴力犯罪，维护良好的社会和经济秩序；推进社会治安、安全生产、环境保护等重点领域信息化建设，提高智能化监管水平，确保社会平安和谐。

　 （六）抓好改革开放。推进重点改革。以组建文化体育旅游集团为牵动，深化文化体制改革；以做好县区公立医院改革试点为切入，深化医药卫生体制改革；以下放事权、创新审批方式为突破口，深化行政管理体制改革；以平稳完成新一轮岗位聘用为着力点，深化事业单位人事管理制度改革。扩大交流合作。加强与航天科技、恒基伟业、清华启迪等强企合作，围绕龙头项目关联吃配组织好重点产业招商，引进超亿元项目50个，到位资金150亿元。抢抓全省建设外贸转型升级示范基地机遇，提升新肇内陆港通关过货能力，集聚发展外贸型企业，全市进出口贸易总值增长20%。营造创业环境。积极引进领军人物、研发团队科技创业，扶持各类经营管理人才、大中专毕业生自主创业，鼓励退转军人、外出务工经商人员返乡创业，大力营造亲民、护商、爱才的优良环境，创办和扶持中小企业800户，全市非公经济增长25%以上。

各位代表，建设现代化国际化城市，各级政府肩负着重大责任和光荣使命。我们将按照市委“两服务”、“两端正”、“两提高”要求，秉承“负责任地干事、干负责任的事”工作理念，从今年开始启动实施以使命宗旨教育为主题、规则意识养成为主线、实际问题解决为突破、行政效能提高为目标、人民群众满意为目的的“政务文化建设工程”，切实加强政府自身建设，更好地适应新形势新任务要求。着力解决思维理念问题。始终以现代化国际化的眼光和思维谋发展搞建设，抓产业项目强化前沿高端意识，抓城市建设强化区域中心意识，抓社会管理强化务实创新意识；始终正视前进道路中遇到的各种挑战和困难，把思维起点放在干事成事上，遇到困难矛盾不说不行，想办法做到怎么能行；始终瞄准先进城市和发达地区，比学赶超，创先争优，推动各项工作站位更高、思路更新、招法更实、进步更快。着力解决群众感情问题。把为大庆人民谋福祉作为推动现代化国际化城市建设的根本目的，坚持发展惠民、务实为民、政务便民、监督靠民、感情亲民，政府决策要进一步倾听民声民意，项目建设要进一步倾向民生事业，公共财政要进一步倾斜民生领域，从一件件具体实事抓起，努力让人民群众最大限度地共享发展成果。着力解决素质作风问题。克服能力不足，抓好学习培训，加强实践锻炼，大力引育人才，不断增强促发展、搞服务、抓落实的真本事硬功夫；克服看摊守业，以只争朝夕的干劲、持之以恒的韧劲、勇担风险的闯劲，突破一个个瓶颈，解决一个个矛盾，攻克一个个难点，不断开创工作新局面；克服敷衍推诿，忠诚履责真正干，不当“二传手”自己干，率先垂范带头干，时不我待抢先干，相互补台合力干。着力解决执行落实问题。在“快”上下功夫，抓落实行动快，抓项目进展快，抓工程进度快，抓发展见效快；在“精”上下功夫，坚决克服“还行”、“可能”、“差不多”等笼统习惯，做到业务精通，标准精细，工作精心，办事精准；在“实”上下功夫，崇尚真抓实干，求实情、出实招、务实效，做好打基础、利长远的事，使发展成果经得起历史和人民的检验。着力解决廉洁从政问题。深入推进依法行政，切实强化规则意识，严格按照法定权限和程序履行职责；进一步深化政务公开和办事公开，确保权力运行依法规范、公开透明；深入落实党风廉政建设责任制，认真执行廉政准则，严肃财经纪律，严控“三公”支出，严禁铺张浪费，树立勤政廉洁的政府形象。

　 始终坚持市委统一领导，主动接受人大法律和工作监督，自觉接受政协民主监督。认真办好人大代表建议和政协委员提案，广泛听取民主党派意见建议，支持工会、共青团、妇联等群团组织开展工作。完善公众参与、专家咨询和政府决定相结合的决策机制，畅通政民互动沟通渠道，努力建设人民满意政府。

　　各位代表，过去的辉煌成就令人鼓舞，未来的宏伟蓝图催人奋进。让我们在市委的坚强领导下，紧紧团结依靠全市各族人民，传承弘扬大庆精神、铁人精神，解放思想、与时俱进、居安思危、团结拼搏，为建设全面小康社会和现代化国际化城市而努力奋斗！
